# Infant feeding and analgesia in labour: the evidence is accumulating

**DOI:** 10.1186/1746-4358-1-25

**Published:** 2006-12-11

**Authors:** Sue Jordan

**Affiliations:** 1 School of Health Sciences, Swansea University, Singleton Park, Swansea, UK

## Abstract

The interesting and important paper by Torvaldsen and colleagues provides further circumstantial evidence of a positive association between intrapartum analgesia and feeding infant formula. Not all research supports this association. Before 'failure to breastfeed' can be adjudged an adverse effect of intrapartum analgesia, the research evidence needs to be considered in detail. Examination of the existing evidence against the Bradford-Hill criteria indicates that the evidence is not yet conclusive. However, the difficulties of obtaining funding and undertaking large trials to explore putative adverse drug reactions in pregnant women may mean that we shall never have conclusive evidence of harm. Therefore, reports of large cohort studies with regression models, as in the paper published today, assume a greater importance than in other areas of investigation. Meanwhile, women and their clinicians may feel that sufficient evidence has accumulated to justify offering extra support to establish breastfeeding if women have received high doses of analgesics in labour.

## Introduction

Torvaldsen and colleagues have published an interesting paper [1] on an extremely important topic: the impact of intrapartum analgesia on infant feeding. If intrapartum analgesics do interfere with breastfeeding, this might, arguably, be the adverse drug reaction with the greatest public health consequences. To explore this issue, Torvaldsen and colleagues revisited a 1997 cohort to examine the factors affecting duration of breastfeeding up to 24 weeks. These authors are among the first to employ a Cox proportional hazards regression model in this field. This illuminating analysis suggests that both intrapartum pethidine and epidurals can increase the likelihood of breastfeeding cessation: 210/292 (72%) women who had no pharmacological analgesia were breastfeeding at 24 weeks compared to 139/261 (53%) who received pethidine and 206/396 (52%) who received epidurals containing bupivacaine and fentanyl (hazard ratios [HR]: 1.95, 95% CI [confidence interval]: 1.45, 2.63 and 2.07, 95% CI: 1.57, 2.72).

## Discussion

Women feed their babies infant formula for a variety of reasons: physical limitations; medical advice; psychosocial factors; cultural norms and expectations. Recently, the possibility that infant feeding is also constrained by pharmacological influences has attracted the attention of researchers. Several studies, including this [1], have suggested that 'failure to breastfeed' is linked to administration of intrapartum analgesics.

When deciding whether to accept, administer or advocate intrapartum analgesia, women, their clinicians, and those who compose clinical guidelines, need to consider whether 'failure to breastfeed' is a foreseeable and preventable adverse effect of opioids or epidurals. Is the association between analgesia and feeding infant formula strong enough to meet the criteria for an adverse drug reaction?

If failure to breastfeed is considered an appreciably harmful or unpleasant reaction, related to the use of intrapartum analgesics, and administration predicts hazard, and warrants prevention, specific treatment, alteration of the dosage regimen, or withdrawal of the product, then this is an adverse drug reaction [2,3]. It is possible that some women, and even some clinicians, will not consider 'failure to breastfeed' an appreciably harmful event, warranting prevention, and here the public health message needs to be communicated. Identification of adverse drug reactions is a socially contingent process. It is possible that infant formula feeding is such a common occurrence that it does not attract the attention of clinicians: in practice, a woman's failure to breastfeed may be more likely to be attributed to cultural norms and expectations than opioid-induced impairment of the suckling reflex. The concern of researchers is to examine the evidence for a causal, rather than a co-incidental, relationship by addressing four pertinent questions [4].

### 1) Is the strength, consistency, specificity and temporality of association between feeding infant formula and intrapartum analgesia sufficiently robust to attribute cause and effect?

Most of the evidence comes from cohort and observation studies. Not all these studies, particularly the small ones, indicate any association [5-8]. These cohorts included 56, 171, 181 and 114 parturients, respectively. No-one has suggested that intrapartum medication is the only determinant of infant feeding, and small cohorts may be unable to allow for the many confounding variables. Other cohort studies, ranging in size from 99 to 411 parturients, have supported the link between epidural analgesia and feeding infant formula [9-13]. With one of the largest cohorts in the area (n = 1178), Torvaldsen and colleagues are making an important contribution to this field [1]. However, even the largest well conducted cohort studies will never provide evidence as robust as that obtained by experimental methods, most particularly, well conducted double-blind randomised placebo-controlled trials.

Intrapartum analgesia is inevitably linked to method of delivery, as this study indicates, and feeding infant formula may be associated with Caesarean birth [14]. There are very few papers reporting the relationship between infant feeding and induction of labour [15], but women whose labour is induced often require more analgesia [16]. Disentangling the contributions of these interacting variables is not always possible. In this study, data were analysed excluding women undergoing Caesarean section, and findings were essentially unchanged. However, variables cannot always be removed from an analysis in this way, which may leave study findings vulnerable to confounding. Even the best statistical modelling cannot entirely compensate for the associations that are unavoidable in clinical practice: only large randomised trials can do this.

### 2) Is there any experimental evidence?

There would be considerable difficulties in allocating women to a placebo arm of a randomised controlled trial, where they would receive no real analgesia during labour. The existing randomised controlled trials in this area are too small and vulnerable to cross-over to fully test the hypothesis that intrapartum analgesia affects infant feeding. Interpretation of the largest trial is confused by the high proportion of women crossing over between the two arms of the trial: 314/499 (62.9%) women from the no-epidural arm required epidurals and 117/493 (23.7%) randomised to receive epidurals did not request them [17]. Per protocol analysis of the subset of 484 women with spontaneous labour and vaginal birth linked epidural analgesia containing fentanyl plus bupivacaine with shorter duration of breastfeeding (HR: 1.44, 95% CI: 1.04, 1.99; p = 0.029). A smaller trial randomised 177 women who had previously breastfed and were requesting epidurals to: bupivacaine only, 1–150 mcg fentanyl or >150 mcg fentanyl [18]. Cross-over between trial arms was 15/177 (8.5%), and reporting of infant feeding at six weeks was complete for 157/177 (89%) women. The findings support an association between administration of epidural fentanyl and feeding infant formula: babies were exclusively formula-fed by 1/51 women who received 0–100 mcg fentanyl, 3/54 women who received 20–350 mcg fentanyl, 10/52 who received 75–350 mcg fentanyl (p = 0.002).

### 3) Is there a dose-response relationship?

A dose-dependent relationship between medication administered and feeding or behaviour has been reported [13,18,19]. However, several researchers, including Torvaldsen and colleagues, were unable to obtain information on doses administered, thus limiting the options for data analysis.

### 4) Is the association biologically plausible and coherent?

All opioids administered to the mother pass into the neonate via both the placenta and the colostrum, but transfer is more rapid and complete for the more lipophilic compounds, such as diamorphine, fentanyl and fentanyl derivatives. The impact on neonatal behaviour is observable and dose-related [19]. The evidence from this paper would suggest that opioids affect infant feeding, regardless of route of administration. This is discussed more fully elsewhere [13].

Labour without recourse to pain relief, whether pharmacological or non-pharmacological, is not an option. If it is considered that, on balance, the evidence supports a causal association, extra support will need to be offered to the most vulnerable women, to ensure that their infants are not disadvantaged by this hidden, but far-reaching, adverse drug reaction. The experience of Henderson and colleagues indicates that undertaking a trial in this area is likely to present many practical difficulties [17], beyond the control of even the most expert researchers. Meanwhile, the absence of data from large successful trials increases the importance of cohort studies, such as that published today [1].

## Conclusion

Clinicians basing their practice on the best available evidence, and women considering their analgesic choices, may feel that, despite the paucity of randomised controlled trials in this area, strategies for mitigating the impact of intrapartum medications on the next generation should be considered. Most particularly, women receiving high doses of opioids might be offered extra support to establish and maintain breastfeeding, coupled with information to help them gain an understanding of some of the reasons underlying any difficulties they are encountering.

## Competing interests

The author(s) declare that they have no competing interests.
